# CEQer: A Graphical Tool for Copy Number and Allelic Imbalance Detection from Whole-Exome Sequencing Data

**DOI:** 10.1371/journal.pone.0074825

**Published:** 2013-10-04

**Authors:** Rocco Piazza, Vera Magistroni, Alessandra Pirola, Sara Redaelli, Roberta Spinelli, Serena Redaelli, Marta Galbiati, Simona Valletta, Giovanni Giudici, Giovanni Cazzaniga, Carlo Gambacorti-Passerini

**Affiliations:** 1 Department of Health Sciences, University of Milano-Bicocca, Monza, Italy; 2 Department of Neurosciences and Biomedical Technologies, University of Milano-Bicocca, Monza, Italy; 3 Tettamanti Research Center, University of Milano-Bicocca, San Gerardo Hospital, Monza, Italy; Institut Jacques Monod, France

## Abstract

Copy number alterations (CNA) are common events occurring in leukaemias and solid tumors. Comparative Genome Hybridization (CGH) is actually the gold standard technique to analyze CNAs; however, CGH analysis requires dedicated instruments and is able to perform only low resolution Loss of Heterozygosity (LOH) analyses. Here we present CEQer (Comparative Exome Quantification analyzer), a new graphical, event-driven tool for CNA/allelic-imbalance (AI) coupled analysis of exome sequencing data. By using case-control matched exome data, CEQer performs a comparative digital exonic quantification to generate CNA data and couples this information with exome-wide LOH and allelic imbalance detection. This data is used to build mixed statistical/heuristic models allowing the identification of CNA/AI events. To test our tool, we initially used in silico generated data, then we performed whole-exome sequencing from 20 leukemic specimens and corresponding matched controls and we analyzed the results using CEQer. Taken globally, these analyses showed that the combined use of comparative digital exon quantification and LOH/AI allows generating very accurate CNA data. Therefore, we propose CEQer as an efficient, robust and user-friendly graphical tool for the identification of CNA/AI in the context of whole-exome sequencing data.

## Introduction

Copy number alterations (CNA) are common events occurring in leukaemias and solid tumors. Comparative Genome Hybridization (CGH) is presently considered the gold standard technique for CNA analysis. However, CGH requires dedicated instruments and it is able to perform only low resolution Loss of Heterozygosity (LOH) analyses. Recently, the development of high-throughput sequencing instruments able to generate hundreds of Gigabases per run allowed the development of completely new approaches to the analysis of cancer genomes [1], [2]. Among them, whole-exome sequencing has been extensively used in the last few years to analyze the coding regions of cancer genomes in order to detect the presence of somatic variants. In a recently published paper [3], however, Lonigro and colleagues formally demonstrated that exome sequencing data can be also used to perform CNA analyses. Subsequently, several bioinformatics pipelines dedicated to the analysis of exome data have been proposed [4]–[8]. These tools proved to be efficient in detecting copy number variations, however they typically require complex command line commands, lack a graphical interface, require complex installation procedures, generally relying on multiple dependencies, and run on costly server-sized machines. Moreover, these tools report no or limited information about allelic imbalance (AI) events (Table 1). Therefore, no graphical, user-friendly bioinformatics tools dedicated to the coupled CNA/AI analysis of exome sequencing data are actually available. To overcome this limitation we developed CEQer (Comparative Exome Quantification analyzer), a new, graphical, event-driven tool for CNA/AI-coupled analysis of exome sequencing reads. By using case-control matched exomes, CEQer generates CNA data through a comparative digital exonic quantification and then couples this information with exome-wide LOH and AI detection. This data is used to build mixed statistical/heuristic models allowing the identification of CNA/AI events at exome level. CEQer runs on standard 32 or 64 bit desktop/notebook PC and accepts the most widely used alignment/pileup file formats (Pileup/BED, SAM and BAM) as input. Being totally graphical and event driven, it requires no a priori bioinformatics or scripting knowledge. It manages either single or multiple jobs by using a dedicated batch tool and generates interactive graphical views as well as textual reports. CEQer was tested using a large set of *in silico* and real exome data: the results are presented here.

**Table 1:** Summary of the main characteristics of 6 copy number analysis tools.

Software	OS	GUI	Language	External dependencies	Input	Interactive Output	Real-time Filters/Settings	Detects
ExomeCopy	L/W/M	NO	R	YES	BAM	NO	NO	CNV
ExomeCNV	L/W/M	NO	R	YES	Coverage	NO	NO	CNV/LOH
CoNIFER	L	NO	Python	YES	RPKM	NO	NO	CNV
ExomeDepth	L/W/M	NO	R	YES	BAM	NO	NO	CNV
VarScan2	L/W/M	NO	JAVA	NO^2^	Pileup	NO	NO	CNV/LOH
CEQer	L/W/M^1^	YES	C#	NO^3^	SAM/BAM/ Pileup	YES	YES	CNV/AI/ LOH

L/W/M: Linux/Windows/MacOS.

1 L/W/M for processing. W is required for visualization and real-time filtering.

2 The Java Runtime is required.

3 The. NET framework Runtime is required. If not present in the system, it is automatically installed during CEQer setup.

## Materials and Methods

### Ethics statement

All the investigations were performed in accordance with the principles embodied in the declaration of Helsinki. Bone marrow (BM) or peripheral blood (PB) samples were collected after written informed consent. The study was specifically approved by the San Gerardo Hospital (Monza, Italy) ethics committee.

### Algorithms

CEQer bioinformatics algorithms (Figure S1 in file S1) can be divided into two main groups: 1) Static algorithms extracting raw coverage and LOH/AI data from input files: these steps are typically performed when new exome data is processed. 2) Real-time algorithms processing raw data from step 1 by applying user-defined filters and parameters (detailed description in the ‘Real-time algorithms for CNAs detection’ section) on-the-fly, generating CNA and LOH/AI data and displaying them in graphical modules.

#### Static algorithms for raw coverage and LOH/AI preprocessing

CEQer accepts either Pileup/BED, SAM or BAM files as input. As a first step, positions in the input case and control files are scanned in order to identify all the exonic bases. To increase the efficiency of this step, data is processed using parallel programming techniques. The specific coverage of each exonic position is calculated by using algorithms optimized for the different input files and using a dedicated exonic database. During this step exons are automatically identified and annotated using the information stored in the database. The use of SAM and BAM files leads to a computational overhead, required to generate pileups for each exonic position starting from individual read coordinates and, limited to BAM, to decompress BGZF file sections. Positions and coverage are stored in a dedicated dictionary object. After the completion of the first step, this object is analyzed in order to generate three different datasets: 1) Mean coverage of each exon; as a direct measure of the internal variability of each exonic count, a per-exon coverage standard deviation is also computed. 2) Whole-exome case and control median and mean coverage. Median coverage will be subsequently used to perform coverage normalization between case and control datasets. Mean and median information for case and control are stored in dedicated files. 3) During the analysis of the control data, ‘raw’ heterozygosity candidates, initially defined as positions where more than a single base is present with a fixed coverage of ≥5, are stored in a dedicated dataset. Case coverage information pertaining to all the exonic positions for the 4 bases are initially saved in a temporary file and then filtered according to the control set. This paired case/control dataset is subsequently used to call the heterozygous positions using dedicated statistical tests and heuristic algorithms, as described in the next section. The static filters available in this section are: 1) ‘Mean Read Quality Filter’, allowing to set a Phred-based read quality threshold applied to the mean quality of individual reads; 2) Individual Base Quality Threshold, allowing to define a per-base read quality threshold; 3) Maximum number of low quality bases, which sets the number of bases with an Individual Base Quality Threshold lower than a user defined threshold that are accepted in each read. Filter 1 and 3 are only applied to read-centered input data, such as SAM and BAM files. Filter 2 is applied also to Pileup files, where each position is tested individually.

#### Real-time algorithms for CNAs detection

After the completion of the initial step or whenever the user loads a previously preprocessed CEQer analysis, real-time algorithms process raw data, identify CNAs and LOH/AI according to user-defined filters and parameters and display data.

The case/control coverage of each exon is initially normalized using the median whole-exome coverage generated during the previous step. The normalized coverage is then used to calculate the case/control coverage ratio and the Log_2_ Ratio. In-depth analysis of each step can be performed using dedicated modules allowing the user to visualize all the phases of the analysis, comprising pre-normalized, normalized, ratio and Log_2_ Ratio exonic views.

In order to detect the CNA regions, CEQer uses a combined statistical and heuristic approach, where normalized case and control data are initially compared using a non-parametric Wilcoxon matched-pairs test.

Let *N* indicate the number of data pairs; 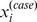 and 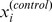 indicate the measurements. For each pair, (1) is calculated.
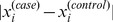
(1)


All the pairs where 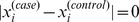 are discarded. All the remaining pairs (*N_r_*) are then sorted in ascending order according with (1) and ranked (*R_i_*) starting from 1. Pairs with identical score are assigned a rank equal to the average of the ranks they span. The test statistic *W* is calculated as follows:
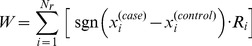
(2)


As the size of N increases, the distribution of W approximates a normal distribution. When *N_r_*>10, the approximation is close enough to calculate a z-ratio with Yates continuity correction as follows:
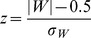
(3)Where *σ_w_*, the standard deviation of the normal distribution, is calculated according to (4).



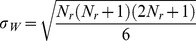
(4)Under this approximation, CEQer calculates the p-value associated with the z-ratio by estimating the error function (5)
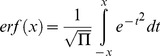
(5)of the normal distribution of *W* using the Abramowitz and Stegun approximation equation 7.1.26 [9], as indicated in (6),
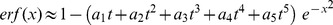
(6)where a1 = 0.254829592; a2 = −0.284496736; a3 =  1.421413741; a4 = −1.453152027; a5 =  1.061405429; q = 0.3275911; 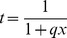.

The p-value is finally calculated by estimating the error function at 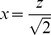.

If *N_r_* ≤10, then the p-value is calculated using an exact sampling distribution by enumerating all the possible combinations of *W* given *N_r_*.

The main advantages of this approach are 1) that differences in the pull-down and sequence efficiency among different exons are intrinsically eliminated by initially performing matched comparisons between identical case and control exons and 2) that no a priori exon coverage distribution must be assumed. Statistical analyses are performed on sliding exonic windows of user-defined length. However, instead of using individual windows as static placeholders for CNA regions, regions with significant p-values are used as ‘seed’ sequences: after the identification of all the CNA seeds, CEQer tries to expand the CNA area on both ends and performs further matched-pairs statistical tests, as described, to check whether the difference in the normalized case/control coverage of the expanded CNA area is still significant. If two seed regions are successfully expanded over two contiguous exons, the two regions are automatically merged in a single CNA. CNA identification is one of the most critical algorithms, therefore CEQer exposes a wide set of filters and parameters to control this process. Specifically, the following user-defined filters/parameters can be applied:

1. CNA p-value: it allows to set a specific threshold p-value for CNA identification.
2. Window size: this parameter controls the size of the seeding window. Smaller windows require slightly longer computational times and are ideal to identify short CNAs.
3. Coverage Correction Factor (CCF): CEQer adds the CCF to all the case/control normalized exonic coverage data before calculating the coverage ratio. It is used to smoothen the effect of low coverage data, however setting very high CCF will decrease the overall sensitivity of the statistical test.
4. Coverage Filter: can be used to filter out exons characterized by a very low coverage. If either the normalized case or control coverage falls below the threshold, both case and control data is discarded. Although useful to smoothen low coverage data, it can potentially cover up the presence of two-copies deletions.
5. Relative Exon Standard Deviation Filter (RESDF): during the preprocessing step, exonic coverage is calculated together with the intra-exonic coverage standard deviation, which is used to measure how variable is the coverage distribution within individual exons. The RESDF filter allows to discard exons whose standard deviation is higher than a defined threshold.
6. Standard Deviation Filter (SDF): CEQer internally calculates the overall standard deviation of individual chromosomes as a surrogate measure of the intrinsic signal noise. The SDF is expressed as standard deviation-fold threshold. CNA candidates whose mean falls below the threshold are discarded. The SDF is very useful to filter noisy data in order to detect short CNA regions, however it may reduce the sensitivity to large CNAs, because the presence of large abnormalities may increase the background standard deviation.

Although the full filter set is available to the end-users, to increase the easiness of the process, CEQer is also able to auto-select all the filters/parameters based on the expected CNA size, which allows to maximize the detection of very small, small, medium and large CNAs (3–20, 20+, 100+, 500+ exons).

#### Real-time algorithms for LOH/AI detection

Raw control heterozygosity candidates, generated by using a fixed coverage of ≥5 for at least two different bases and stored in the temporary control dataset, are initially parsed in order to retrieve the coverage of each base. All the positions in the prefiltered control dataset are individually processed to identify the two most frequent bases for each position. The information pertaining the remaining bases is discarded. Subsequently, Goodness-of-fit statistical tests modeling a perfect heterozygosity are generated for each candidate in order to identify control positions compatible with an heterozygous status. The whole set of bona fide heterozygous positions are then tested in the matched case dataset. For each position, the two most frequent bases in the case dataset are annotated together with their coverage; the information pertaining the other bases is discarded. New Goodness-of-fit statistical tests are performed to check whether the heterozygosity is conserved in the case sample. If the relative coverage of the two case alleles is compatible with an heterozygous status, the position is deemed to be in ‘conserved heterozygosity’ and the information is stored in a dedicated object. If the coverage distribution of the two bases in the case exome does not fit with an heterozygous distribution, then the position is considered a LOH/AI. To further characterize the LOH/AI status, the coverage of the case LOH/AI position is initially normalized according to the whole-exome median case/control coverage and then compared to the corresponding control heterozygous position by using fixed thresholds: in presence of a case coverage ≤0.75 fold the control coverage, the LOH/AI event will be considered as a putative ‘Copy Number Loss’; between 0.75 and 1.25 as a putative ‘Copy Neutral Loss’; between 1.25 and 1.75 as a ‘+1 Copy Number Gain’; over 1.75 as a ‘+2 or more Copy Number Gain’. CEQer can optionally process LOH/AI data by applying a sliding window smoothing algorithm whose size is controlled by a Window Size parameter. It can be used in presence of noisy LOH/AI data to identify real LOH regions.

### Data visualization

CNA and LOH/AI are shown in real-time in dedicated visualization modules. To allow a realistic visualization, the position of each exon is proportional to its real position in the genome.

Several views are available to facilitate data analysis and quality control. Specifically, the whole-exome View, where the Log_2_Ratios of all the chromosomes are shown in a single panel, allows the user to directly inspect the whole dataset and to quickly move to individual chromosomes. Among the chromosome views the most critical is the Log_2_Ratio View, where the Log_2_Ratio of individual chromosomes is shown. Here, the CNA regions are plotted and parameters/filters can be set to refine the analysis and the results in real-time. LOH/AI data can be visualized in the same view, which allows to easily link CNA and LOH/AI information. LOH/AI view can be fully customized to show only specific LOH events, such as ‘Copy Number Loss’, ‘+1 Copy Gain’, ‘+2 or more Copy Gain’, ‘Copy Neutral Allelic Imbalance’ and Conserved Heterozygous positions, custom combination of them or the whole LOH/AI set. To allow a thorough, in-depth analysis of exome data, CEQer also generates Raw, Normalized and Ratio Coverage views. Finally, a Whole-Exome Coverage View reports critical information about case and control exonic coverage, thus allowing to easily detect coverage imbalance problems and facilitating the generation of quality reports.

All views can be saved as raster or vector files and data from each view can be also exported as tabular files. Many visualization parameters, such as background color and gradient style, exon size and color, LOH/AI and CNA markers are fully customizable. Each view can be freely zoomed-in and out to focus on specific regions; complete information about single exons and LOH/AI loci are shown as tooltip text.

### Generation of in silico data

To perform extensive *in silico* simulations, a test-generator (CopyNumberTester) has been developed. This tool generates CNA data by simulating the output of a standard CEQer preprocessing step. By using this approach, data generated by CopyNumberTester can be directly processed by CEQer. In our models, copy number was calculated according to the following equation:
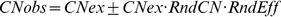
(7)where *CNobs* represents the final copy number value, as stored in the output file; *CNex* is the expected, theoretical copy number value in absence of any stochastic effect, *RndCN* is a user-defined, positive floating-point factor defining the maximum extent of the copy number variability and a second factor *RndEff* is a random floating-point ranging from 0 to 1 and controlling the real extent of each random effect. The activation of a CNA event is controlled by a set of 3 user-defined probability factors for +/−1, +/−2, and >2 copies CNA events.

*In silico* LOH/AI data is controlled by the following equation:
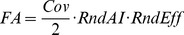
(8)where *FA* represents the coverage of the first allele at heterozygous positions; *Cov* models the overall coverage, *RndAI* is a user-defined, positive floating-point factor defining the maximum amount of stochastic noise in the calculation of the allelic ratio and *RndEff* is defined as in CNA equation.

### Exome Sequencing

All the exome libraries were generated from 1 µg gDNA extracted with Invitrogen PureLink Genomic DNA (gDNA) Kit (Invitrogen, Life technology, Grand Island, NY, USA). Only non-degraded gDNA (A260/280 ratio between 1.8 and 2.0 and A260/230 ratio >2.0) was used. gDNA was fragmented to a size of 500–100 bp using a Bandelin Sonopuls sonicator (Cycles: 50; Processing time: 10 sec; Pulsation: 20%; Power (amplitude): 10%) and then processed according to standard Illumina TruSeq^TM^ DNA Sample Preparation Kit protocol, with selection of a 200–300 bp fragment on 2% agarose gel. Multiplexed genomic libraries were then pooled and enriched for exome sequences using two rounds of hybridizations with capture probes of target regions provided in the Illumina TruSeq^TM^ Exome Enrichment Kit. The libraries were subsequently sequenced on an Illumina Genome Analyzer IIx with 76 bp paired-end reads using Illumina TruSeq^TM^ SBS kit v5.

### Accession codes

High-throughput sequencing data have been deposited in the Sequence Read Archive (SRA) under accession SRA096103.

### CEQer

CEQer is implemented entirely in C# under the. NET framework v.4.0. The static algorithms, which represent the most time consuming steps, can be run on 64/32 bit Windows, Linux or Mac (under Mono: http://www.mono-project.com/CSharp_Compiler) operative systems; all the real time and visualization steps require a 64/32 bit Windows operative system (successfully tested under Windows 7, Vista and XP). CEQer has been designed using streaming and parallel programming technologies requiring a limited memory footprint. It runs on standard dual or quad core, 4 Gbytes memory desktop/notebook PC. The typical timing required to complete an analysis using 80× coverage Pileup datasets as input is 50 minutes on a 4 Gbytes, QuadCore Intel i7 X 940 Notebook.

### Patients

Leukemic cells were obtained by separation on a Ficoll-Paque Plus gradient (GE Healthcare, UK) from BM or from the buffy coat fraction of PB samples followed by the lysis (155 mM NH_4_Cl, 10 mM KHCO_3_ and 0.1 mM EDTA) of erythrocytes. The phenotype was evaluated by FACS analysis. As source of normal cells, samples obtained after complete cytogenetic remission or lymphocytes obtained culturing cells with 2,5 µg/ml Phytohemagglutinin-M (PHA-M, Roche- Switzerland) and 200 UI/ml Interleukin-2 (IL-2, Aldesleukin, Novartis – Switzerland) for 3–4 days followed by 2–3 weeks incubation with IL-2 only were used. The phenotype was evaluated by FACS analysis and lymphoid cells (CD3, CD4, CD5, CD8, CD19) resulted to be >80% of total cells.

### Array CGH Analysis

Genomic copy number analysis was performed by a-CGH using an Agilent Human Genome CGH Microarray 2×400 K kit (Agilent Technologies^TM^, Santa Clara CA, United States) following the manufacturer's recommendations. The microarray contains 411,056 probes with 5.3 Kb overall median probe spacing (4.6 Kb for Refseq genes). Target and reference DNA were extracted from the same patient, cancer tissue (target) was hybridized against normal tissue (reference). Target DNA was labeled with cyanine Cy3 (emission in green fluorescence) and reference DNA was labeled with cyanine Cy5 (emission in red fluorescence). The analysis was performed using Feature Extraction v10.7 and DNA Analytics v6.5 software (Agilent Technologies^TM^, Santa Clara CA, United States) applying ADM2 algorithm with a threshold of 5, minimum absolute average log2 ratio in called intervals of DLRS (Derivative Log2Ratio Spread) value and a minimum of 3 consecutive probes. Putative chromosome copy number changes were defined by intervals of 3 or more adjacent probes and were considered as being duplicated or deleted when results exceeded the ±DLRS-value range.

All nucleotide positions were referred to the Human Reference Sequence Assembly Mar 2006 NCBI36/hg18 of UCSC (http://genome.ucsc.edu/).

### Fluorescent In Situ Hybridization

Cytogenetic investigation was carried out on bone marrow cells according to standard methods; metaphases were treated for QFQ banding and analyzed using epifluorescent microscope and Macktype 5.5.4 (PSI, US); chromosomal abnormalities were defined using the recommendations of the International Systems for Human Cytogenetic Nomenclature (ISCN 2005). Interphase fluorescent in situ hybridization (FISH) analysis was performed using locus specific probe LSI BCR/ABL Dual color Dual Fusion Probe (Abbott Molecular, Illinois, US) according to manufacturer's instructions; a total of 100 nuclei were counted.

### PCR-based Sanger sequencing

100 ng of genomic DNA were amplified with FastStart Taq DNA Polymerase (Roche-Applied-Science, Germany) according to manufacturer instructions. The primers used for the amplification were: rs7021384 – *Chr9-132543814-Fw* TGT GGA GGG GTG CTC AGA GAC, *Chr9-132543814-Rw* CCT CTC TGC CTC TCT CAT TCT CTC; rs16936946 – *Chr9-131609353-Fw* GCT CTG ACC TCG TCC TTC AGT G, *Chr9-131609353-Rw* GGC TTG ATT GCG TCA ATG ATC; rs735115 – *Chr9-131677533-Fw* CTC ACC CCA CAG GAA GAG CAG, *Chr9-131677533-Rw* GTG GGA GCA GAG GAA GGT CTG; rs1503375 -*Chr9-131663114-Fw* AGC CCC TCT GTT CAC TGT TTT C, *Chr9-131663114-Rw* GGT TTC CAG CCT CCA GAT GAG; rs11792431 -*Chr9-131555068-Fw* GAC GTG GAG AGA AGC AAG TAT GAC, *Chr9-131555068-Rw* CCC CTC CCA CTT TGT GCT TTC; rs74487784 – *Chr22-22655095-Fw* GGAACTTGTCCTCCAGCCATTG, *Chr22-22655095-Rw* CCAGGTGAGGAGAGCCATCTG. Amplified products were run on agarose gels, purified with QIAquick Gel extraction kit (Qiagen Sciences, USA) and sequenced through standard Sanger sequencing. Sequences were analyzed using Vector NTI 7.0 (Invitrogen, Carlsbad, CA, US).

## Results

To assess the ability of our tool to detect CNA and AI events, a wide set of *in silico* exome data was initially generated and analyzed. Subsequently, CNA/AI analyses were performed in the context of real cancer exomes. The main advantages of this approach over a direct analysis of patient specimens are that 1) by using *in silico* modeled data, the exact number of CNA loci and the characteristics of each event are *a priori* known; therefore, reliable tests can be generated. 2) Fine tuning of each model can be performed by modulating critical parameters, such as case and control coverage, background and allelic ratio noise, length and frequency of each CNA locus.

### In silico analyses

To assess whether CEQer is able to correctly identify and report the presence of large CNA regions, we generated a first CNA model where we enforced the presence of copy number alterations of +/−1 exon with a fixed CNA size of 80 exons. In order to accurately assess CEQer ability to identify CNA events, a total of 10 tests were run. A correct identification for this and the following tests was considered whenever CEQer identified a CNA region with at least 80% region overlap with the modeled event. In this simulation, CEQer identified all the abnormalities (158/158), either copy gain or loss, scoring 100% sensitivity and 100% positive predictive value, with no false positive events (Table S1 in file S1). We subsequently modeled +/−1 CNA events of 10 and 4 exons. In line with the first analysis, we ran 10 simulations per each test. In the ‘10 exons’ simulations a total of 170 CNAs were generated. Of them, 165 were correctly identified by CEQer (true positive), with an overall sensitivity of 97.1%. Only 2 false positive events were reported, with a positive predictive value of 99% (Table S1 in file S1). In the ‘4 exons’ simulations, a total of 145 CNAs were generated. Of them, 112 tested positive, with a sensitivity of 77.2%. No false positive events were reported, with a positive predictive value of 100% (Table S1 in file S1). Similar results were achieved with a ‘3 exons’ simulation, were a total of 163 CNA events were generated and 142 correctly detected (sensitivity: 87.1%; positive predictive value: 98%).

A critical issue when analyzing copy number data is that, in presence of large CNA regions, the predicting algorithms may become less reliable, leading to phenomena such as CNA hypersegmentation, were large individual CNA events are erroneously segmented into smaller fragments. To assess whether CEQer is able to correctly identify and report the presence of chromosome-sized CNA regions, we ran 10 simulations using a new CNA model where we enforced the presence of a total of 105 extremely large CNA events with a mean length of 8000 exons. In presence of these conditions, CEQer detected all the CNA regions (Table S1 in file S1) with 100% sensitivity, no false positives and in absence of hypersegmentation.

To further put our tool under test in a more complex scenario, we modeled the co-existence of long (80 exons) and short (4 exons) CNA events in a set of 4 new simulations (Table S1 in file S1). A total of 22 events was modeled; of them, 11 were long and 11 short CNAs.

In 3 out of 11 long CNAs, evidence of a limited hypersegmentation was detected: the CNA region was split into two subregions comprising, in total, over 90% of the original CNA. Globally, CEQer identified 21/22 events, with a sensitivity of 95% and a positive predictive value of 88%.

### Coverage imbalance

When whole-exome sequencing is used to identify copy number variants, ‘case’ sequencing data is normalized using a matched control. This allows to compensate for the effects of inter-exon pull-down and sequencing efficiency and of germline indels. However, due to specific experiment design (e.g. lower coverage of the control dataset) or to fluctuations of the whole-exome sequencing quality, the coverage of the matched case and control exomes can be largely different, which may in turn impair our ability to efficiently use exome sequencing for CNA detection. To assess the effect of highly imbalanced case/control coverage, we generated two new models where we enforced a 4× case/control coverage ratio in favor of the case or of the control, respectively, and in presence of 10-exons CNA events. Ten simulations per model were generated, with a mean exonic coverage of 100 and 25 for case and control (T100H25: 86 events in total) or *vice versa* (T25H100: 93 events in total). Coverage reports generated by CEQer (Fig. S2a, b in file S1) allowed to easily identify the presence of highly imbalanced exomes. In the T100H25 and T25H100 tests, a significant coverage drop was detected by CEQer in the control or case exome, respectively, at 25× (Fig. S2b in file S1), as expected. In line with coverage reports, raw per-exon coverage data confirmed the presence of highly imbalanced data (Fig. S2c in file S1; chromosome 2 is shown as example). To deal with coverage imbalance, CEQer performs a whole-exome median coverage normalization (see Materials and Methods for details), whose effect is reported in the normalized case/control counts report window (Fig. S2d in File S1) and in the case/control ratio window (Fig. S2e in file S1) for in-depth data analysis. Despite the presence of highly imbalanced exome data, analysis of the T100H25 dataset (Fig. S2f and g in file S1: chromosome 2 and whole genome view of simulation #1 are shown as example) using CEQer led to the identification of 71 CNAs, with a sensitivity of 82.6% and a 100% positive predictive value (Table S1 in file S1). The same analysis on T25H100 led to the detection of 90/93 real events, with an overall sensitivity of 96.8% and a positive predicting value of 97% (Table S1 in file S1).

### Allelic imbalance

One of the critical advantages of exome sequencing-based copy number analyses over CGH is that it is possible to co-detect CNA events together with AI/LOH. To analyze AI events, CEQer initially scans the control sequences in order to generate a map of all the heterozygous positions throughout the exome. Subsequently, the heterozygosity of these positions is tested in the ‘case’ exome by using a combined statistical and an heuristic approach which takes into account the allelic ratio of case and control samples, the median case/control exonic coverage and the absolute total coverage at the specified nucleotide position (see Materials and Methods for details). Using this approach, CEQer analyzes each heterozygous position and outputs a predicted case allelic status among: ‘Conserved Heterozigosity’, ‘Loss of 1 or 2 allele (s)’, ‘Loss of Heterozigosity with conserved copy number’, ‘Gain of 1 allele’ and ‘Gain of 2 or more alleles’. Notably, CEQer automatically merges the AI/LOH analysis together with the CNA data to facilitate the integration of the combined information. Thanks to the optimization of the analytical procedures, CEQer is able to compute the effect of all the CNA and AI/LOH filters in real-time, which allows the end-user to perform an extensive fine-tuning of all the experiment settings.

To test the ability of our tool to detect AI/LOH events, we modeled a new set of *in silico* experiments where we generated CNA events involving, on average, 200 exons and a mean case and control exonic coverage of 100×. We modeled the effect of CNA events in terms of predicted allelic imbalance, enforcing an increasing stochastic effect (*RndAI*, see Materials and Methods for details) on the coverage of the two alleles. To model the real effect of the allelic ratio variability, the stochastic effect was not limited to the CNA regions but extended to all the heterozygous positions throughout the exome, which allowed also to assess the specificity of our tool in discriminating between real AI/LOH and spurious imbalances due to the stochastic variability of the allelic counts. The *RndAI* parameter was initially set at 0.05 and then increased up to 0.30. Five *in silico* tests per each *RndAI* setting were generated. The analysis was limited to a single chromosome (Chr4) where a total of 3 CNA events were modeled: a single copy deletions, a +1 and a +2 copy gain. A total of 22 heterozygous positions were present in the 3 CNA regions: 10 in the −1, 6 in the +1 and 6 in the +2 CNA. The ability to discriminate among the different AI events is dependent on three factors: 1) the heterozygous positions calls occurring in the control dataset, 2) the AI calls occurring in the case dataset and 3) the ability to correctly categorize each AI event. The sensitivity of the heterozygous calling algorithm in the 3 CNA regions was 97.8% at 0.05 *RndAI* and 97.6% at 0.30 (Fig. S3a in file S1). No false positive heterozygous calls were detected in the 3 CNA regions (Table. S1 in file S1). The AI calling algorithm was very sensitive in presence of low background noise (95.6% with 0.05 *RndAI*), with 100% detection of single copy loss and +2 copy gain events and 86.7% of the +1 gain (Fig. S3b in file S1). In presence of high background noise (0.3), the overall sensitivity was 65.1%, with 62% detection of the single copy loss, 83.3% of the +2 copy gain and 50% of the +1 gain. No false positive AI calls were detected in the 3 CNA regions (Table S1 in file S1). AI categorization was correct in 100% of the single copy loss and of the +2 copy gain and in 86.7% of the +1 gain at 0.05 *RndAI*, while it dropped to 46, 83.3 and 50% at 0.3 *RndAI* (Fig. S3c in file S1).

### In silico analyses using real whole-exome data

We then sought to simulate very small CNA events in the context of real exonic data. We used matched whole-exome data from patient aCML-007 (Table S2 in File S1), where no evidence of copy number events could be detected. We artificially generated simulations of 4-exons duplications/deletions occurring in either chromosome 1 or in chromosome X. Ten CNA events per simulation were generated; of them on average 5 were duplications and 5 deletions. Data analysis for chromosome 1 simulations revealed that, on a total of 40 CNA events, CEQer was able to correctly identify 29, with an overall sensitivity of 72.5%. A total of 4 regions were erroneously identified as copy number abnormalities (false positive), with a positive predictive value of 88% (Table S1 in file S1). The same analysis performed on chromosome X led to the identification of 36/40 events, with an overall sensitivity of 90%. A total of 3 regions were erroneously identified as CNAs (false positive), with a positive predictive value of 92% (Table S1 in file S1).

### Patient samples

We then sought to verify whether our tool was similarly able to identify CNA events using real whole-exome data. Therefore, a total of 40 exomes were generated from 20 leukemic specimens and corresponding matched controls (Table S2 in file S1). Of them, 12 were from classical Chronic Myeloid Leukemia (CML) and 8 from Atypical Chronic Myeloid Leukemia (aCML) [10] patients. Among the CML patients, 9 were in chronic phase (CP) at onset of the disease and before any leukemia-related treatment and 3 were in blast crisis (BC). According to the literature [11], the genome of chronic myeloid neoplasms is characterized by a limited chromosomal instability. The opposite occurs in BC, where the genome of the leukemic cells is typically unstable [12].

**Figure 1:**
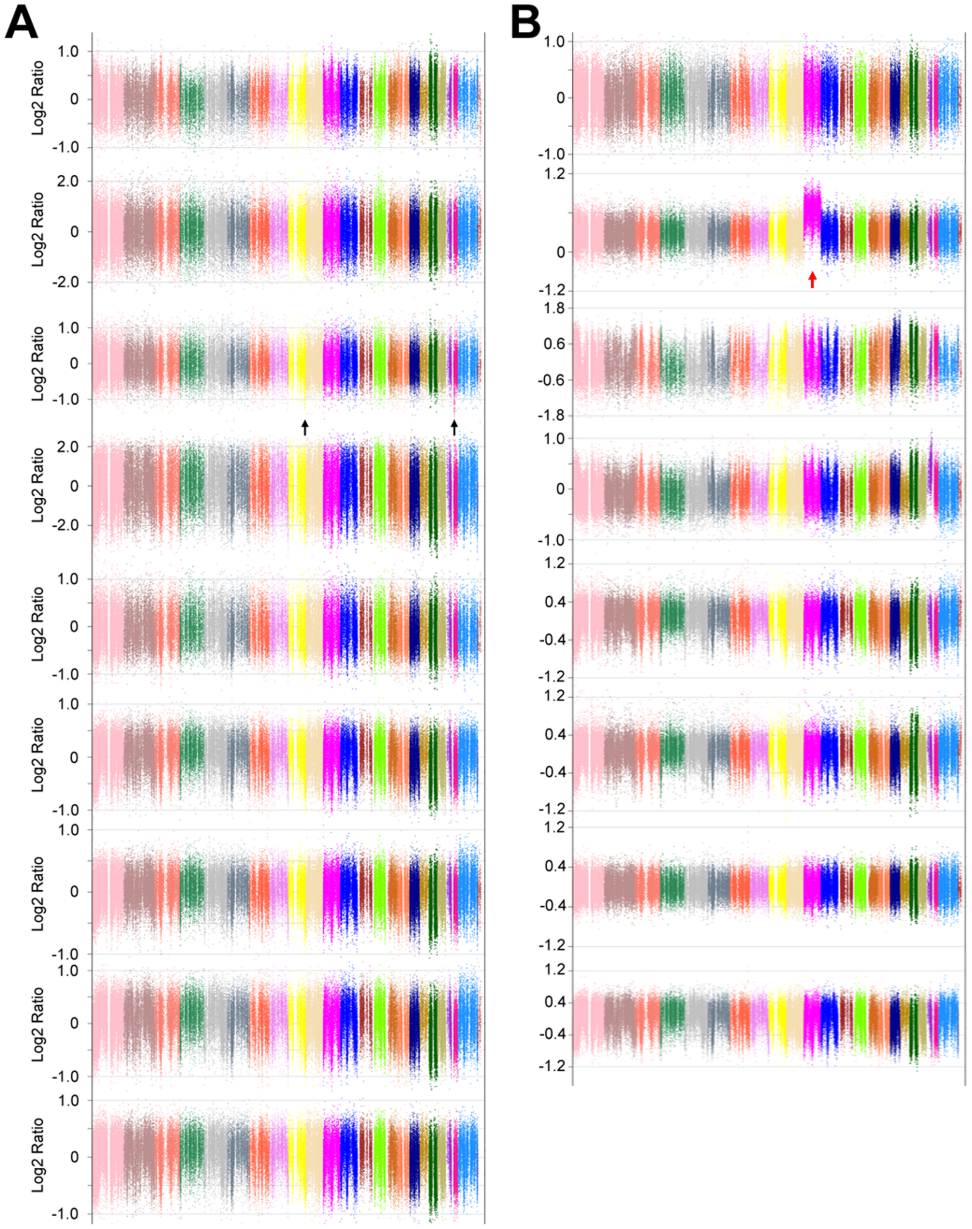
Whole-genome view of CML-CP (a, CML-CP-001 to 009) and aCML samples (b, aCML-001 to 008). The two black arrows point to a deletion occurring in patient CML-CP-003; the red arrow points to an amplification of the whole chromosome 11 in patient aCML-002.

**Figure 2:**
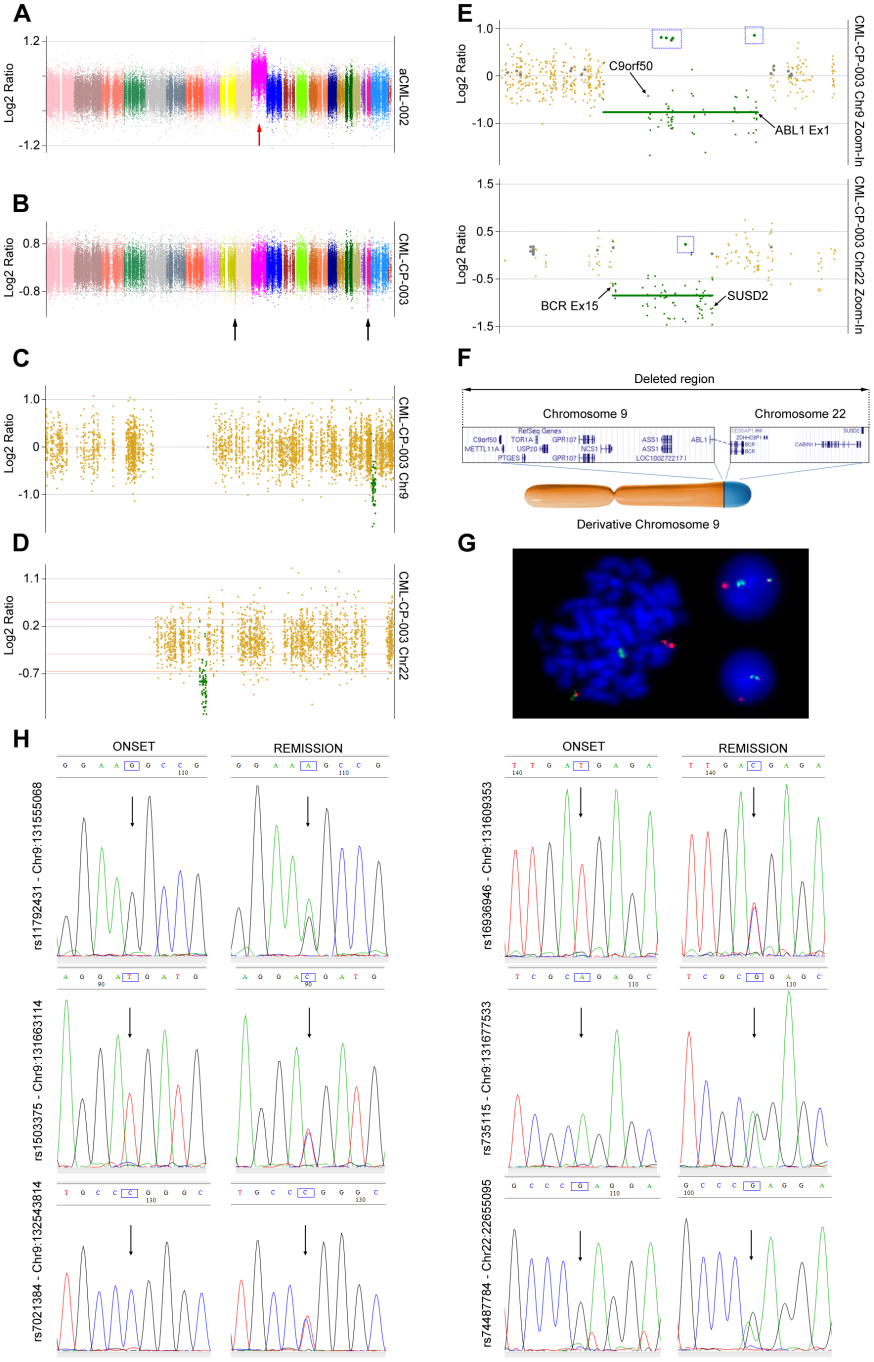
CNA analysis of sample aCML-002 and CML-CP-003. Whole-exome view of patients aCML-002 (a) and CML-CP-003 (b). Coverage data are indicated as Normalized Log2 Ratios. Individual colors represent specific chromosomes from 1 (left) to Y (right). Red and black arrows indicate CNA areas in patient aCML-002 and CML-CP-003, respectively. c, d) Individual views of chromosome 9 (c) and 22 (d) of patient CML-CP-003. Dark-yellow dots indicate copy neutral exons, green dots copy loss CNA exons. Thick green horizontal bars identify copy loss regions. e) Detailed view of the boundaries delimiting the CNA regions in chromosome 9 (upper panel) and 22 (lower panel). Individual genes/exons delimiting the copy loss CNA region (s) are shown. Blue boxed rectangles highlight the candidate LOH positions, marked as green circles. f) Proposed model of derivative chromosome 9 partial deletion: the boxed regions represent the deleted genomic loci as identified by CEQer. The relative position of the two regions in the derivative chromosome 9 is shown in the bottom part of the panel. g) Dual color *BCR/ABL1* FISH analysis of patient CML-CP-003 at onset of the disease. h) Sanger sequencing at onset of the disease and upon cytogenetic remission of 6 heterozygous single nucleotide polymorphism sites shown in panel e, identified by CEQer as putative LOH loci. The blue boxes indicate the heterozygous nucleotides; the black arrows point to the corresponding chromatogram peaks.

On average 9 Gigabases were generated for each exome, with a mean exonic coverage of 80x. As expected, the analysis of CML-CP and aCML patients revealed the presence of a limited number of CNAs (Fig. 1). Specifically, copy number events could be detected (Fig. 2a, b) only in one aCML (aCML-002) and one CML patient (CML-CP-003). Sample CML-CP-003 was particularly interesting, because a previous analysis using FusionAnalyser [13] revealed that in the leukemic cells a loss of the reciprocal *ABL1-BCR* gene in the derivative chromosome 9 (der9) was present. However, cytogenetic analysis failed to reveal the expected loss of the complete der9 (data not shown), suggesting the presence of a cryptic deletion involving only the *ABL1-BCR* locus. In this patient, two copy number loss events were detected by CEQer, apparently involving chromosome 9 and 22 (Fig. 2c, d). In depth analysis of these events using CEQer (Fig. 2e) revealed that chromosome 9 deletion occurred between gene *C9orf50* and the first coding exon of *ABL1*. Chromosome 22 deletion involved a locus comprised between *BCR* exon 15 and the *SUSD2* gene. Notably, *ABL1* exon 1 and *BCR* exon 15 are the two breakpoint exons most frequently involved in the *ABL1-BCR* reciprocal fusion occurring in der9. Taken together, this data suggested the presence of a cryptic fusion occurring within the derivative chromosome 9, spanning from gene *C9orf50* to *SUSD2* and comprising the whole *ABL1-BCR* fusion gene (Fig. 2f). In line with this data, FISH analysis confirmed the loss of the *ABL1-BCR* gene (Fig. 2g) in der9. CGH analysis using a 400 k Agilent Human Genome CGH Microarray as well as genomic Q-PCR (not shown) experiments confirmed the presence of the copy number loss (Fig. S4 in file S1), as indicated by CEQer. Notably, the application of the LOH/AI detection algorithms allowed the identification of a total of 6 candidate Loss of Heterozygosity positions within the CNA locus, 5 in the *C9orf50*-*ABL1* and 1 in the *BCR*-*SUSD2* region of the der9 (Fig. 2e). All the genomic positions corresponding to the 6 LOH/AI candidates were identified as Single Nucleotide Polymorphism loci (SNPs, rs11792431, rs16936946, rs1503375, rs735115, rs7021384 and rs74487784, respectively) according to the dbSNP135 database [14], suggesting that the heterozygous calling algorithm of CEQer was robust enough to reliably detect heterozygous positions in the control exome. To verify if the somatic LOH/AI predictions were correct, the genomic loci corresponding to the 6 LOH/AI candidates were amplified and sequenced in the case and matched control: as expected, direct analysis of the sequenced amplicons confirmed the presence of heterozygosity in the controls and of somatic LOH in the leukemic samples for all the 6 candidates (Fig. 2h).

**Figure 3:**
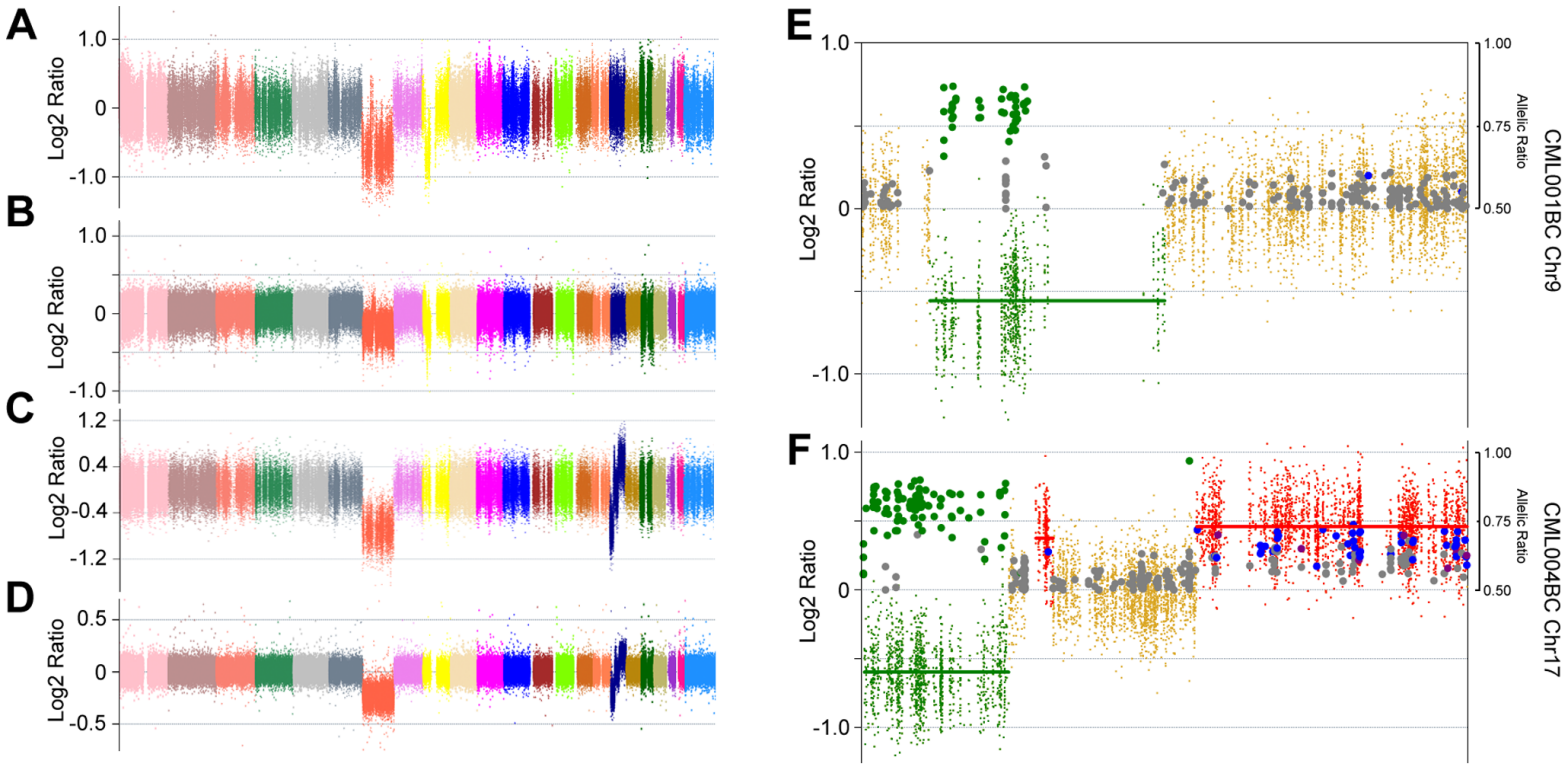
CNA analysis of sample CML001BC and CML004BC. a) Genome view of patient CML001BC analyzed by CEQer and (b) corresponding CGH analysis. c) Genome view of patient CML004BC and (d) corresponding CGH analysis. Genome views of coverage data are represented as Normalized Log2 Ratios. Individual colors represent specific chromosomes from 1 (left) to X (right). e) Individual views of CML001BC-chromosome 9 and of CML004BC-chromosome 17. Dark-yellow dots indicate copy neutral exons; green dots copy loss and red dots copy gain CNA regions. Thick red and green horizontal bars identify copy gain and loss regions, respectively. Large gray circles indicate heterozygous positions in the control dataset whose heterozygosity is conserved in the case. Large green, blue and dark-red circles indicate copy loss, +1 copy gain and +2 copy gain LOH/AI events, respectively. The position of LOH/AI markers on the y axis indicates the corresponding allelic ratio.

**Figure 4:**
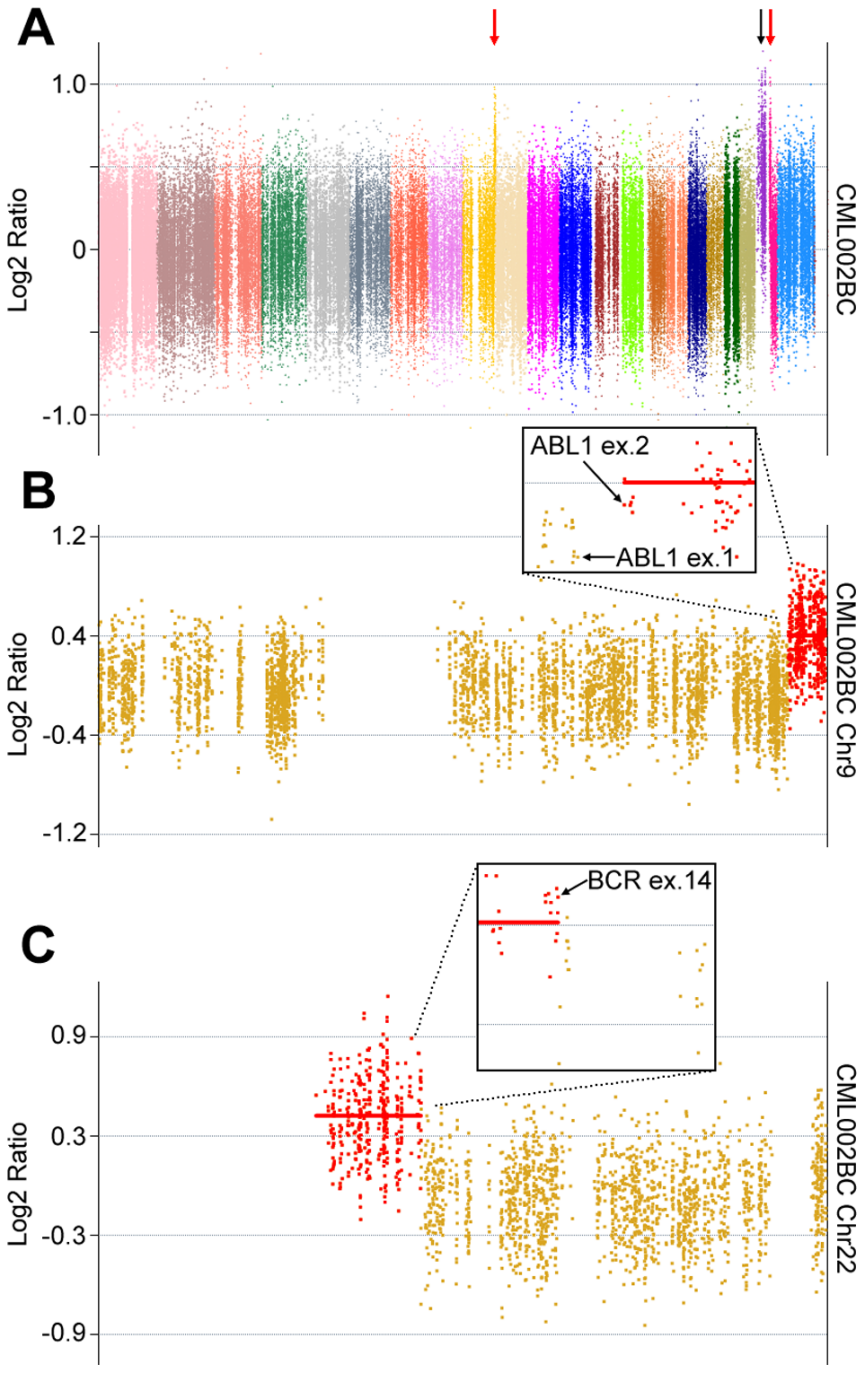
CNA analysis of sample CML002BC. a) Genome view of patient CML002BC. Coverage data are represented as Normalized Log2 Ratios. Individual colors represent specific chromosomes from 1 (left) to Y (right). b) Individual views of chromosome 9 and chromosome 22 (c). Dark-yellow dots indicate copy neutral exons, red dots copy gain CNA regions. Thick red horizontal bars identify copy gain regions. Boxed panels represent magnified views of the CNA boundaries: individual genes/exons delimiting the copy gain CNA region (s) are indicated.

In line with previous reports, indicating that chromosomal instability increases upon progression from CP-CML to BC [12], CEQer analysis of three matched BC/CP exomes revealed the presence of large CNA events occurring in all the samples (Fig. 3 and 4). In two cases, a large (CML001BC) or complete (CML004BC) deletion involving chromosome 7 was detected (Fig. 3a, c). This was not unexpected, because partial or complete single-copy loss of chromosome 7 is one of the most frequent event detected upon progression to BC [15]. In sample CML001BC, the loss of chromosome 7 was accompanied by a partial loss of a large pericentromeric region of chromosome 9, spanning 54 Mbases and involving the *CDKN2A/p16* ^Ink4a^ oncosuppressor. In accordance with CNA data, LOH/AI analysis on chromosome 9 (Fig. 3e) revealed the presence of a large cluster of ‘copy number loss’ LOH events occurring within the deleted region.

In sample CML004BC, the complete loss of chromosome 7 was accompanied by a complex set of CNA events occurring in chromosome 17 (Fig. 3f). Here, a large deletion, spanning 19.2 Mbases and involving the vast majority of the short arm of chromosome 17, comprising the locus of the *TP53* oncogene, was detected. This region was followed by a copy neutral, pericentromeric region spanning 24.3 Mbases and by a 35.1 Mbases copy gain region, comprising almost the whole long arm of chromosome 17. Within the copy neutral pericentromeric region, a second, relatively short copy gain region spanning 2.0 Mbases and extending from the *WSB1* to the *CRYBA1* gene was reported. In line with CNA data, LOH/AI analysis revealed a pattern of ‘copy number loss’ LOH within the deleted region, while a cluster of ‘single copy gain’ AI events was correctly reported in the copy gain regions (Fig. 3f). CGH analysis performed on the same samples revealed a virtually identical CNA pattern (Fig. 3b, d).

In patient CML002BC, together with a duplication of the whole chromosome 21, two apparent copy gains were detected, occurring in chromosome 9 and 22 (Fig. 4a). In depth analysis of these events revealed that chromosome 9 copy gain started at *ABL1* exon 2 and involved the whole distal fraction of chromosome 9 long arm (Fig. 4b); chromosome 22 copy gain involved the whole short arm and pericentromeric regions and ended at *BCR* exon 14 (Fig. 4c). Notably, chromosome 9 and 22 CNA start and end were identical to the breakpoint exons of the CML002BC *BCR-ABL1* fusion and the genes involved in the copy gain abnormality perfectly superimpose with the genes present in the Philadelphia chromosome, suggesting that the two CNAs are instead a single copy gain of the whole Philadelphia chromosome comprising the *BCR-ABL1* fusion oncogene.

To formally compare the reliability of our tool in detecting CNA regions with CGH analyses, we built a dedicated CEQer module where the linear correlation between CEQer and CGH data, expressed as case/control ratios in user-defined windows spanning throughout the whole genome (chromosome Y is excluded from the analysis), can be tested using a Pearson product-moment correlation analysis (see Materials and Methods for further information). As an ideal candidate for this test we used the CML004BC sample, where significant areas of copy number gain and loss were present. In this context, the correlation between CEQer and CGH was very high (Pearson's *r*  = 0.96, Fig. S5 in file S1), suggesting a close, direct, linear relationship between CEQer and CGH data. As may be expected, applying the same test to individual CNA or copy neutral regions led to a Pearson's *r* of near 0 (data not shown). This suggests that the strong linear relationship between exome and CGH data holds true when regions with different copy number are tested, while the distribution of exome and CGH case/control ratio data within individual CNA or copy neutral regions is largely governed by stochastic factors with weak or no direct relationship.

Finally, we sought to compare CEQer with already available CNA software. One of most widely used CNA tools with LOH detection capabilities is VarScan2 [8]. Although VarScan2 is a very powerful software for somatic variants detection, its ability to perform CNA/LOH analyses is still limited. Specifically: 1) VarScan2 only takes into account LOH; no other allelic imbalances are computed. 2) LOH are calculated only for positions where Case and Control don't match so they give limited contribution to the interpretation of CNA data. 3) No allelic imbalance information is available for heterozygous positions. 4) Homozygous/Heterozygous discrimination is performed by using a 75% fixed threshold: no statistical test (s) supporting each evidence is performed. 5) VarScan2, as many other bioinformatics tools dedicated to high-throughput sequencing analyses, performs calculations using a set of user defined filters. However, changing filters and parameters requires rerunning the whole analysis, which is very time expensive when dealing with Gbyte-sized data. CEQer instead performs an initial set of pre-calculations, storing ‘intermediate data’ as output files and then performs filtering and calculations in real-time, allowing to easily test multiple filtering strategies. 6) VarScan2 calculates individual Log2Ratio data but has very limited clustering support (http://varscan.sourceforge.net/using-varscan.html#v2.3_copynumber).

To test VarScan2 performance and compare it with CEQer, we applied the VarScan2 ‘copynumber’ module using the default settings to the CML001BC and CML004BC pileup samples as indicated in the VarScan2 manual: both analyses required approximately 2 hours using VarScan2 and 50–55 minutes (respectively) with CEQer using identical hardware. At the end of the analysis, VarScan2 generated a textual output, reporting a total of 1278654 and 1196137 copy number segments, respectively. Focusing on CML001BC and to restrict the analysis only to the most relevant events, we filtered the VarScan2 report to CNA putative events with at least 40 consecutive segments with the same Log_2_ Ratio sign. Even with this stringent approach, a considerable number of CNA events were reported (636). As expected, the majority clustered in chromosome 7 (465; 73.1%) and chromosome 9 (125; 19.6%), suggesting that VarScan2 hypersegmented the large CNA events occurring in these two chromosomes. Surprisingly, a large number (46) of copy number events was also reported for chromosome 1, 2, 3, 5, 8, 11, 14, 15, 16, 17, 21, 22, X and Y. None of them were identified by CEQer or reported in CGH analyses (Fig. 3b, d) suggesting that, at least with standard settings, VarScan2 had good sensitivity but limited specificity. Taken together, these data show that CEQer is faster and more specific than VarScan2. Moreover, the use of VarScan2 requires further downstream processing to convert segment data into biologically meaningful results.

### CEQer and Circos

Whole-genome copy number data as well as other events, such as fusions, point mutations and indels, are commonly reported as circular diagrams using Circos [16]. To facilitate the upload of CEQer data into Circos, a dedicated CEQer function has been built. By using it, CEQer converts CNA data into the Circos format, so that they can be smoothly imported for circular diagram generation. To generate diagrams, together with properly formatted data, Circos requires a complex configuration file. To ease the process of circular diagram creation, whenever the conversion function is triggered, together with the Circos-formatted CNA data, CEQer automatically generates a dedicated configuration file which can be directly imported in Circos with no further manual editing. Taking into account the fact that exome sequencing data is typically used to identify single nucleotide variants/small indels together with CNAs, CEQer automatically generates a commented-out section of the configuration file pointing to an optional mutation file.

## Discussion

Similarly to RNA-Seq, where transcript abundance can be assessed by analyzing individual sequences as digital counts, in whole-exome sequencing individual case/control reads aligning to specific exons can be used to estimate the relative copy number of the corresponding genomic locus. Although this possibility has been clearly demonstrated [3] and despite the large diffusion of exome sequencing as a standard approach to characterize genetic lesions in cancer, its use as a source of CNA and LOH/AI information is very limited. To overcome these limitations, we developed CEQer. To allow our tool to be run on standard desktop/notebook computers, CEQer has been implemented with an extensive use of streaming technologies, parallel programming and memory efficient algorithms. This is not trivial because many sequencing companies are now offering exome sequencing and primary analysis (i.e. Fastq generation, alignment and quality report) at competitive costs: therefore, the use of CEQer allows CNA/LOH analyses to be potentially performed even in laboratories where costly bioinformatics infrastructures and high-throughput sequencers are not available and in absence of any dedicated bioinformatics support.

To further simplify CNA and LOH/AI analytical pipelines, a flexible input module has been built, allowing CEQer to automatically accept a wide variety of input file formats, such as the Pileup/BED, SAM and BAM formats, thus eliminating the need of running complex command line scripts or file format conversion tools to preprocess input data; analyses can be run individually or as serial jobs using a dedicated graphical batch tool; full analytical settings can be stored as parameter files and reloaded in new analyses; whole-exome and individual chromosome data are exportable as textual files for further processing or as images; views can be freely zoomed to analyze genomic regions in greater detail and exported as raster or vector files; after the preprocessing step, filters and parameters can be freely modified in real-time, with the new output generated within few seconds using standard 4 Gb RAM, two or quad-core notebook or desktop computers. All CEQer views, filters and parameters are controlled by using simple *point-and-click* procedures.

To validate our tool we extensively tested it by using a large set of *in silico* generated copy number models, with variable length and frequency of the CNA events and variable case/control coverage and with a set of 20 matched exomes from leukemic samples and we compared the results with gold standard techniques such as the CGH analysis. In virtually all the conditions tested our tool proved to efficiently detect CNA variants and LOH/AI events, allowing the identification of a significant number of previously unreported copy number lesions. Taken globally, these data suggest that CEQer is an efficient and easy-to-use graphical tool for CNA/AI detection in the context of exome-sequencing experiments.

CEQer, databases, documentation and test files are available for download from: http://www.ngsbicocca.org/html/ceqer.html.

## Supporting Information

File S1 **Figure S1, Flowchart of the main CEQer algorithms. Figure S2, CEQer analysis of** ***in silico*** **generated CNA data, modeling imbalanced whole-exome data (sample T25H100, simulation #1). Figure S3, Results of the** ***in silico*** **analysis of allelic imbalance data. Figure S4, Whole-genome view of the copy number analysis performed in sample CML-CP-003 using CEQer (a) and CGH (b). Figure S5, Linear correlation between the whole-exome Case/Control exonic ratios as generated by CEQer (x axis) and CGH (y axis) for CML004BC sample. Table S1, Results of the** ***in silico*** **tests. Table S2, Summary of clinical details of the patients included in this study.**
(DOCX)
