# Supplementary material for: CEQer: A Graphical Tool for Copy Number and Allelic Imbalance Detection from Whole-Exome Sequencing Data
Source: PLoS One. 2013 Oct 4;8(10):e74825. doi: 10.1371/journal.pone.0074825 (PMC3790773; doi:10.1371/journal.pone.0074825)
Supplement: File S1 — Figure S1, Flowchart of the main CEQer algorithms. Figure S2, CEQer analysis of in silico generated CNA data, modeling imbalanced whole-exome data (sample T25H100, simulation #1). Figure S3, Results of the in silico analysis of allelic imbalance data. Figure S4, Whole-genome view of the copy number analysis performed in sample CML-CP-003 using CEQer (a) and CGH (b). Figure S5, Linear correlation between the whole-exome Case/Control exonic ratios as generated by CEQer (x axis) and CGH (y axis) for CML004BC sample. Table S1, Results of the in silico tests. Table S2, Summary of clinical details of the patients included in this study. (DOCX) [file pone.0074825.s001.docx]

Suppl. Fig. S1. Flowchart of the main CEQer algorithms. The light grey cylinders represent non-volatile storage systems; the dark grey cylinder indicates the internal exon database. The dotted rectangle indicates the real-time algorithms.


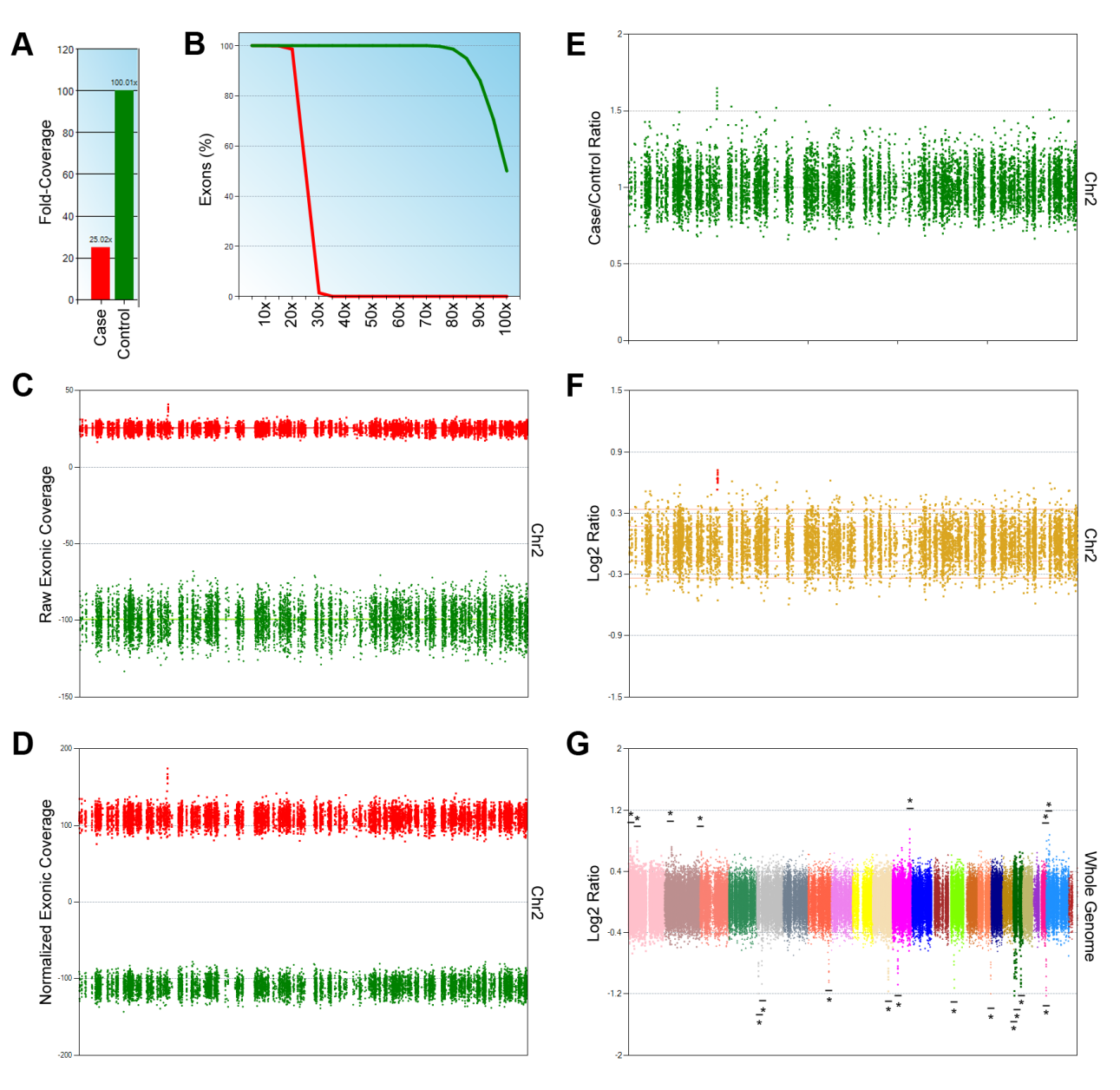


Suppl. Fig. S2. CEQer analysis of *in silico* generated CNA data, modeling imbalanced whole-exome data (sample T25H100, simulation #1). a) Coverage reports. The red and green bars show the mean exonic coverage of case and control dataset, respectively. b) The red and green curves show the percentage of exons (y axis) reaching a specific coverage (x axis). c) Representative Raw exonic coverage (chromosome 2): the red and green dots show the raw coverage of case and control exons, respectively. Control coverage is reported in the negative hemiaxis. d) Normalized exonic coverage for chromosome 2: the red and green dots show the normalized coverage of case and control exons, respectively. Control coverage is reported in the negative hemiaxis. e) Case/control coverage ratio. Green dots report the value of each case/control exon coverage ratio. f) Normalized Log2 Ratio for chromosome 2. Dark-yellow dots indicate copy neutral exons, red dots copy gain CNA regions, as identified by CEQer. g) Whole-genome view of coverage data represented as Normalized Log2 Ratios. Individual colors represent specific chromosomes from 1 (left) to Y (right). Horizontal black lines highlight the presence of *in silico* generated CNA regions. Asterisks indicate candidate CNA regions as identified by CEQer.


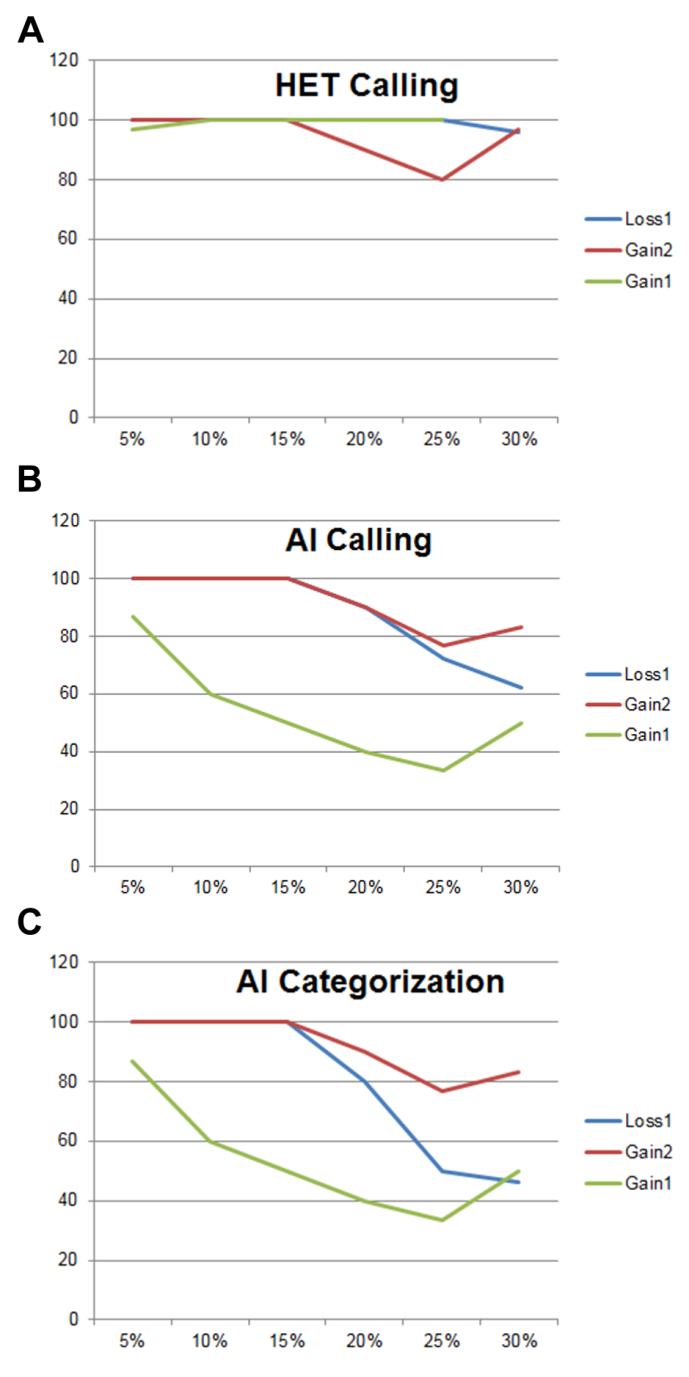


Suppl. Fig. S3. Results of the *in silico* analysis of allelic imbalance data. a) Percentage of correct heterozygous calling in control datasets. b) Percentage of correct AI calling in case datasets. c) Percentage of correct AI categorization in case datasets. -1, +1 and +2 events are represented as blue, green and red lines, respectively.


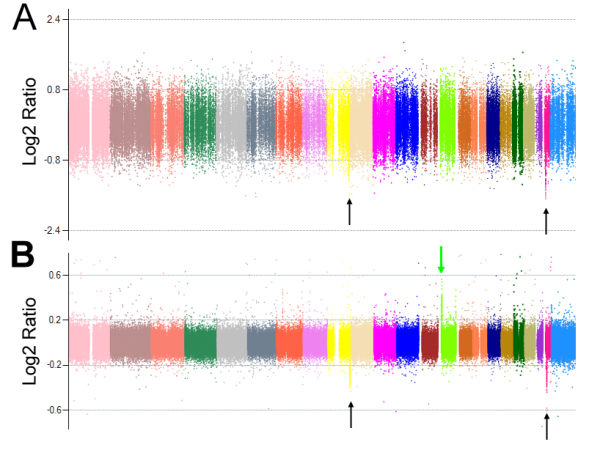


Suppl. Fig. S4. Whole-genome view of the copy number analysis performed in sample CML-CP-003 using CEQer (a) and CGH (b). The black arrows point to a deletion occurring in chromosome 9 and 22; the green arrow points to an intergenic amplification.


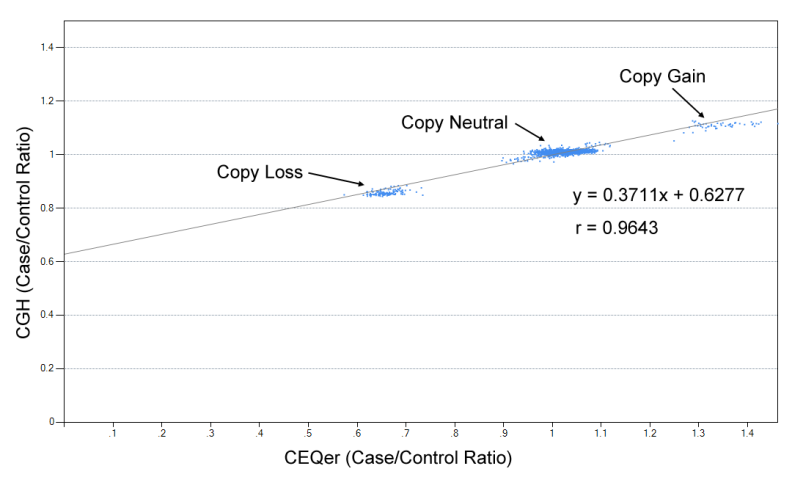


Suppl. Fig. S5. Linear correlation between the whole-exome Case/Control exonic ratios as generated by CEQer (x axis) and CGH (y axis) for CML004BC sample. The value of the Pearson correlation coefficient *r* and the regression equation are shown.

Suppl. Tab. S1. Results of the *in silico* tests.

| In silico: 80 exons CNA | | | | | | | | | | |
| --- | --- | --- | --- | --- | --- | --- | --- | --- | --- | --- |
|  | Sim. 1 | Sim. 2 | Sim. 3 | Sim. 4 | Sim. 5 | Sim. 6 | Sim. 7 | Sim. 8 | Sim. 9 | Sim. 10 |
| T+ C+ | 15 | 15 | 18 | 15 | 13 | 12 | 20 | 12 | 20 | 18 |
| T- C+ | 0 | 0 | 0 | 0 | 0 | 0 | 0 | 0 | 0 | 0 |
| T+ C- | 0 | 0 | 0 | 0 | 0 | 0 | 0 | 0 | 0 | 0 |
| T- C- | - | - | - | - | - | - | - | - | - | - |

|  |  | CONDITION | |  |  |
| --- | --- | --- | --- | --- | --- |
|  |  | True Positive | True Negative |  |  |
| TEST OUTCOME | Test Positive | 158 | 0 | 100 | Positive Predictive Value |
|  | Test Negative | 0 | - | - | Negative Predictive Value |
|  |  | 100 | - |  |  |
|  |  | SENSITIVITY | SPECIFICITY |  |  |

| In silico: 10 exons CNA | | | | | | | | | | |
| --- | --- | --- | --- | --- | --- | --- | --- | --- | --- | --- |
|  | Sim. 1 | Sim. 2 | Sim. 3 | Sim. 4 | Sim. 5 | Sim. 6 | Sim. 7 | Sim. 8 | Sim. 9 | Sim. 10 |
| T+ C+ | 13 | 18 | 21 | 15 | 15 | 9 | 19 | 14 | 21 | 20 |
| T- C+ | 2 | 0 | 0 | 0 | 0 | 1 | 1 | 0 | 0 | 1 |
| T+ C- | 0 | 0 | 1 | 0 | 0 | 0 | 1 | 0 | 0 | 0 |
| T- C- | - | - | - | - | - | - | - | - | - | - |

|  |  | CONDITION | |  |  |
| --- | --- | --- | --- | --- | --- |
|  |  | True Positive | True Negative |  |  |
| TEST OUTCOME | Test Positive | 165 | 2 | 99 | Positive Predictive Value |
|  | Test Negative | 5 | - | - | Negative Predictive Value |
|  |  | 97.06 | - |  |  |
|  |  | SENSITIVITY | SPECIFICITY |  |  |

| In silico: 4 exons CNA | | | | | | | | | | |
| --- | --- | --- | --- | --- | --- | --- | --- | --- | --- | --- |
|  | Sim. 1 | Sim. 2 | Sim. 3 | Sim. 4 | Sim. 5 | Sim. 6 | Sim. 7 | Sim. 8 | Sim. 9 | Sim. 10 |
| T+ C+ | 9 | 5 | 10 | 11 | 9 | 10 | 15 | 13 | 15 | 15 |
| T- C+ | 3 | 4 | 3 | 4 | 2 | 5 | 2 | 2 | 5 | 3 |
| T+ C- | 0 | 0 | 0 | 0 | 0 | 0 | 0 | 0 | 0 | 0 |
| T- C- | - | - | - | - | - | - | - | - | - | - |

|  |  | CONDITION | |  |  |
| --- | --- | --- | --- | --- | --- |
|  |  | True Positive | True Negative |  |  |
| TEST OUTCOME | Test Positive | 112 | 0 | 100 | Positive Predictive Value |
|  | Test Negative | 33 | - | - | Negative Predictive Value |
|  |  | 77.24 | - |  |  |
|  |  | SENSITIVITY | SPECIFICITY |  |  |

| 80 exons + 4 exons | | | | |
| --- | --- | --- | --- | --- |
|  | Sim. 1 | Sim. 2 | Sim. 3 | Sim. 4 |
| T+ C+ | 3 | 7 | 5 | 6 |
| T- C+ | 0 | 0 | 0 | 1 |
| T+ C- | 0 | 2 | 0 | 1 |
| T- C- | - | - | - | - |

|  |  | CONDITION | |  |  |
| --- | --- | --- | --- | --- | --- |
|  |  | True Positive | True Negative |  |  |
| TEST OUTCOME | Test Positive | 21 | 3 | 88 | Positive Predictive Value |
|  | Test Negative | 1 | - | - | Negative Predictive Value |
|  |  | 95.45 | - |  |  |
|  |  | SENSITIVITY | SPECIFICITY |  |  |

| In silico: 8000 exons CNA | | | | | | | | | | |
| --- | --- | --- | --- | --- | --- | --- | --- | --- | --- | --- |
|  | Sim. 1 | Sim. 2 | Sim. 3 | Sim. 4 | Sim. 5 | Sim. 6 | Sim. 7 | Sim. 8 | Sim. 9 | Sim. 10 |
| T+ C+ | 14 | 10 | 9 | 12 | 10 | 10 | 12 | 10 | 8 | 10 |
| T- C+ | 0 | 0 | 0 | 0 | 0 | 0 | 0 | 0 | 0 | 0 |
| T+ C- | 0 | 0 | 0 | 0 | 0 | 0 | 0 | 0 | 0 | 0 |
| T- C- | - | - | - | - | - | - | - | - | - | - |

|  |  | CONDITION | |  |  |
| --- | --- | --- | --- | --- | --- |
|  |  | True Positive | True Negative |  |  |
| TEST OUTCOME | Test Positive | 105 | 0 | 100 | Positive Predictive Value |
|  | Test Negative | 0 | - | - | Negative Predictive Value |
|  |  | 100 | - |  |  |
|  |  | SENSITIVITY | SPECIFICITY |  |  |

| In silico: Coverage Imbalance - T100H25 | | | | | |
| --- | --- | --- | --- | --- | --- |
|  | Sim. 1 | Sim. 2 | Sim. 3 | Sim. 4 | Sim. 5 |
| T+ C+ | 17 | 14 | 10 | 15 | 15 |
| T- C+ | 4 | 2 | 4 | 2 | 3 |
| T+ C- | 0 | 0 | 0 | 0 | 0 |
| T- C- | - | - | - | - | - |

|  |  | CONDITION | |  |  |
| --- | --- | --- | --- | --- | --- |
|  |  | True Positive | True Negative |  |  |
| TEST OUTCOME | Test Positive | 71 | 0 | 100 | Positive Predictive Value |
|  | Test Negative | 15 | - | - | Negative Predictive Value |
|  |  | 82.56 | - |  |  |
|  |  | SENSITIVITY | SPECIFICITY |  |  |

| In silico: Coverage Imbalance – T25H100 | | | | | |
| --- | --- | --- | --- | --- | --- |
|  | Sim. 1 | Sim. 2 | Sim. 3 | Sim. 4 | Sim. 5 |
| T+ C+ | 21 | 16 | 13 | 18 | 22 |
| T- C+ | 1 | 0 | 0 | 0 | 2 |
| T+ C- | 1 | 0 | 1 | 1 | 0 |
| T- C- | - | - | - | - | - |

|  |  | CONDITION | |  |  |
| --- | --- | --- | --- | --- | --- |
|  |  | True Positive | True Negative |  |  |
| TEST OUTCOME | Test Positive | 90 | 3 | 97 | Positive Predictive Value |
|  | Test Negative | 3 | - | - | Negative Predictive Value |
|  |  | 96.77 | - |  |  |
|  |  | SENSITIVITY | SPECIFICITY |  |  |

| In silico/real exomes: Chromosome 1 | | | | |
| --- | --- | --- | --- | --- |
|  | Sim. 1 | Sim. 2 | Sim. 3 | Sim. 4 |
| T+ C+ | 6 | 7 | 7 | 9 |
| T- C+ | 4 | 3 | 3 | 1 |
| T+ C- | 1 | 1 | 1 | 1 |
| T- C- | - | - | - | - |

|  |  | CONDITION | |  |  |
| --- | --- | --- | --- | --- | --- |
|  |  | True Positive | True Negative |  |  |
| TEST OUTCOME | Test Positive | 29 | 4 | 88 | Positive Predictive Value |
|  | Test Negative | 11 | - | - | Negative Predictive Value |
|  |  | 72.50 | - |  |  |
|  |  | SENSITIVITY | SPECIFICITY |  |  |

| In silico/real exomes: Chromosome X | | | | |
| --- | --- | --- | --- | --- |
|  | Sim. 1 | Sim. 2 | Sim. 3 | Sim. 4 |
| T+ C+ | 8 | 10 | 9 | 9 |
| T- C+ | 2 | 0 | 1 | 1 |
| T+ C- | 0 | 1 | 1 | 1 |
| T- C- | - | - | - | - |

|  |  | CONDITION | |  |  |
| --- | --- | --- | --- | --- | --- |
|  |  | True Positive | True Negative |  |  |
| TEST OUTCOME | Test Positive | 36 | 3 | 92 | Positive Predictive Value |
|  | Test Negative | 4 | - | - | Negative Predictive Value |
|  |  | 90.00 | - |  |  |
|  |  | SENSITIVITY | SPECIFICITY |  |  |

Suppl. Tab. S2. Summary of clinical details of the patients included in this study.

| Patient ID | Age at diagnosis | Sex | Sokal score | WBC (*10^3^/µl) | Hemoglobin (g/dl) | Platelets (*10^3^/µl) | Blasts (%) | Karyotype |
| --- | --- | --- | --- | --- | --- | --- | --- | --- |
| CML-CP-001 | 25 | M | 0.8 | 147.4 | 12.7 | 921 | 3 | 10/10 Ph+ |
| CML-CP-006 | 68 | M | 1.6 | 87.2 | 11.1 | 162 | 1 | 20/20 Ph+ |
| CML-CP-005 | 52 | M | 0.6 | 55.7 | 14.2 | 281 | 0 | 20/20 Ph+ |
| CML-CP-003 | 45 | M | 0.9 | 34.4 | 13.5 | 1068 | 0 | 20/20 Ph+ |
| CML-CP-007 | 49 | M | 0.6 | 30.0 | 14.3 | 371 | 0 | 20/20 Ph+ |
| CML-CP-010 | 82 | F | 0.9 | 36.4 | 13.8 | 159 | 0 | 20/20 Ph+ |
| CMLPh+011 | 82 | M | 0.9 | 24.0 | 10.2 | 112 | 0 | 20/20 Ph+ |
| CML-CP-012 | 32 | M | 0.79 | 30.5 | 14.7 | 394 | 0 | 20/20 Ph+ |
| CML-CP-014 | 23 | F | 0.57 | 40.4 | 11.8 | 629 | 0 | 20/20 Ph+ |
| aCML-001 | 75 | M | n.a. | 50.0 | 9.1 | 34 | 9 | No abnormalities |
| aCML-002 | 57 | M | n.a. | 14.3 | 6.9 | 102 | 2 | Chromosome 11 trisomy |
| aCML-003 | 83 | M | n.a. | 23.5 | 11.4 | 314 | 0 | No abnormalities |
| aCML-004 | 49 | M | n.a. | 51.3 | 10 | 338 | 0 | No abnormalities |
| aCML-005 | 74 | M | n.a. | 22.4 | n.a. | n.a. | n.a. | No abnormalities |
| aCML-006 | 75 | M | n.a. | 66.5 | n.a. | n.a. | n.a. | No abnormalities |
| aCML-007 | 65 | M | n.a. | 261.0 | 7.2 | 324 | 1 | No abnormalities |
| aCML-008 | 68 | F | n.a. | 53.9 | 10 | 219 | 5 | No abnormalities |
| CML001BC | 84 | F | 0.92 | 44.4 | 14 | 90 | 85 | 20/20 Ph+ |
| CML002BC | n.a. | F | n.a. | n.a. | n.a. | n.a. | 66 | 20/20 Ph+ |
| CML004BC | 64 | F | 0.93 | 30.0 | 6.9 | 8 | 82 | 20/20 Ph+ |
